# Heme oxygenase-1 reverses HIV-1 Tat activity: prospects for AIDS prevention

**DOI:** 10.1186/1742-4690-9-S2-P221

**Published:** 2012-09-13

**Authors:** ME Cueno, K Imai, K Ochiai

**Affiliations:** 1 Nihon University School of Dentistry, Tokyo, Japan

## Background

Heme oxygenase-1 (HO-1) is a HEME regulator and plays a role in ameliorating HIV-1 infection. In particular, HO-1 inhibits Tat-dependent activation of HIV-1 LTR promoter inhibiting viral gene expression. This suggests that increasing HO-1 activity in HIV-infected cells can reverse Tat action which may contribute to AIDS prevention. However, the correlation between HO-1 and HIV-1 Tat has not been fully elucidated. In order to fully understand how increasing HO-1 activity reverses Tat action and result into the prevention of HIV infection, the mechanism behind the correlation between HIV-1 Tat and HO-1 should first be established.

## Methods

Throughout the study we made use of Jurkat T cells (control) and Jurkat-Tat T cells. Whole cell extracts were obtained and mitochondrial extracts were isolated separately. HO-1, HEME, superoxide dismutase (SOD), catalase and hydrogen peroxide (H2O2) levels were measured using commercially available assays. Immunoassays confirmed both the presence of Tat and NADPH oxidase activity via the HEME-activated gp91phox.

## Results

We found that in Tat-expressing cells, HO-1 and SOD amounts were decreased, HEME and H2O2 levels were increased and catalase concentration was unchanged. In addition, we observed an accumulation of gp91phox and H2O2 amounts. We suspect that Tat activity in Jurkat T cells lead to the following sequence of events: (1) decrease in HO-1 and SOD activities; (2) low SOD amounts leaves catalase amounts unchanged; (3) low HO-1 levels allows HEME to accumulate; (4) high amounts of HEME favors the accumulation of the gp91phox subunit which subseuqently increases NADPH oxidase activity; and (5) ultimately leads to H2O2 accumulation. We hypothesize that by increasing HO-1, as previously reported, HIV-1 infection was ameliorated ascribable to a reversal in Tat activity.

## Conclusion

HIV-1 Tat lowers HO-1 activity which consequentially leads to H2O2 accumulation. We suspect, based on a previous report, that increasing HO-1 ameliorated HIV-1 infection by reversing Tat activity.

